# A Comparison of PBDE Serum Concentrations in Mexican and Mexican-American Children Living in California

**DOI:** 10.1289/ehp.1002874

**Published:** 2011-04-15

**Authors:** Brenda Eskenazi, Laura Fenster, Rosemary Castorina, Amy R. Marks, Andreas Sjödin, Lisa Goldman Rosas, Nina Holland, Armando Garcia Guerra, Lizbeth Lopez-Carillo, Asa Bradman

**Affiliations:** 1 Center for Environmental Research and Children’s Health (CERCH), School of Public Health, University of California at Berkeley, Berkeley, California, USA; 2 Division of Laboratory Sciences, National Center for Environmental Health, Centers for Disease Control and Prevention, Atlanta, Georgia, USA; 3 National Institute of Public Health, Cuernavaca, Mexico

**Keywords:** biomarkers, children, DDE, DDT, flame retardants, human blood serum, human exposure, Mexican, PBDEs, prenatal

## Abstract

Background: Polybrominated diphenyl ethers (PBDE), which are used as flame retardants, have been found to be higher in residents of California than of other parts of the United States.

Objectives: We aimed to investigate the role of immigration to California on PBDE levels in Latino children.

Methods: We compared serum PBDE concentrations in a population of first-generation Mexican-American 7-year-old children (*n =* 264), who were born and raised in California [Center for Health Analysis of Mothers and Children of Salinas (CHAMACOS) study], with 5-year-old Mexican children (*n =* 283), who were raised in the states in Mexico where most CHAMACOS mothers had originated (Proyecto Mariposa).

Results: On average, PBDE serum concentrations in the California Mexican-American children were three times higher than their mothers’ levels during pregnancy and seven times higher than concentrations in the children living in Mexico. The PBDE serum concentrations were higher in the Mexican-American children regardless of length of time their mother had resided in California or the duration of the child’s breast-feeding. These data suggest that PBDE serum concentrations in these children resulted primarily from postnatal exposure.

Conclusions: Latino children living in California have much higher PBDE serum levels than their Mexican counterparts. Given the growing evidence documenting potential health effects of PBDE exposure, the levels in young children noted in this study potentially present a major public health challenge, especially in California. In addition, as PBDEs are being phased out and replaced by other flame retardants, the health consequences of these chemical replacements should be investigated and weighed against their purported fire safety benefits.

Polybrominated diphenyl ethers (PBDEs) are flame retardants used in polyurethane foam, plastics, textiles, and electronics. They are semivolatile, persistent, bioaccumulative compounds that have been produced in three technical preparations named according to their average bromine content: penta-BDE, octa-BDE, and deca-BDE. Penta-BDE and octa-BDE were voluntarily phased out in the United States and Europe in 2004. However, exposure to PBDEs is likely ongoing as these compounds continue to migrate out of products manufactured before this time. Additionally, deca-BDE, which is still used in consumer electronics, wire insulation, and back coatings of draperies and upholstery, may devolve into lower-brominated congeners (Stapleton and Dodder 2008).

Lower-brominated PBDEs are endocrine-disrupting compounds with long half-lives in humans ranging from 2 to 12 years (Geyer et al. 2004). Higher exposure levels are associated in humans with longer time-to-pregnancy (Harley et al. 2010), altered menstrual cycles (Chao et al. 2010), decreased sperm counts (Akutsu et al. 2008), altered thyroid hormone levels in adults (Chevrier et al. 2010; Meeker et al. 2009; Turyk et al. 2008) and infants (Herbstman et al. 2008), and developmental neurotoxicity in children exposed prenatally (Herbstman et al. 2010; Roze et al. 2009).

Because PBDEs are not chemically bound to substrates, they are found in household dust, food and, to a lesser extent, air (Frederiksen et al. 2009; Lorber 2008). Previous studies suggest that ingestion and dermal absorption of house dust is the primary route of PBDE exposure in the United States, particularly among children (Johnson-Restrepo and Kannan 2009; Lorber 2008; Stapleton et al. 2008). It is estimated that up to 91% of the body burden of a breast-fed infant is acquired via breast milk (Johnson-Restrepo and Kannan 2009), but as children begin to interact more with their environment, an increasing proportion of exposure is due to nondietary ingestion of dust resulting from hand-to-mouth contact (Stapleton et al. 2008). Johnson-Restrepo and Kannan (2009) estimated that 77% of body burdens in 1- to 5-year-olds and 58% in 6- to 11-year olds could be attributable to dust. PBDE concentrations in house dust are significantly correlated with levels in breast milk (Wu et al. 2007) and in serum of adults (Johnson et al. 2010).

PBDE serum concentrations are about 20 times higher in the United States than in Europe (Hites 2004; Sjödin et al. 2008a; Zuurbier et al. 2006), with data from the U.S. National Health and Nutrition Examination Survey (NHANES) showing the highest levels in California residents (Zota et al. 2008). Windham et al. (2010) documented higher levels of penta-BDE congeners in 6- to 9-year-old girls from California (*n =* 343) [adjusted geometric mean (GM) of the sum of six congeners: 89.8 ng/g lipid] than in those from Ohio (*n =* 256) (65.9 ng/g lipid); Windham et al. hypothesized that this result was attributable to California’s unique furniture flammability laws. Another study of California children (average age = 3.7 years) with autism (*n =* 50) or developmental delay (*n =* 25) and children from the general population (*n =* 25) found that Hispanic children (*n =* 44) had lower levels of the higher-brominated PBDEs than non-Hispanic children (Rose et al. 2010). They also observed that children of Mexican-born mothers had lower PBDE concentrations than children of U.S.-born mothers. PBDE levels in the children were unrelated to the length of time foreign-born mothers had lived in the United States; however, only 13 of these foreign-born mothers were from Mexico. Currently, more than half of California’s children (52%) are born to Latina mothers (California Perinatal Profiles 2009), of whom most are of Mexican origin. More than half of these Mexican-American children are born to immigrant parents (U.S. Census Bureau 2009).

In this analysis, we compared blood concentrations of PBDEs in two large groups of children of Mexican origin: *a*) 264 7-year-old Mexican-American children born and raised in California, and *b*) 283 5-year-old children born and raised in the Mexican states from which most of the mothers of the U.S.-born children had emigrated. We also measured blood concentrations of two other persistent organic pollutants: *p,p*´-dichlorodiphenyltrichloroethane (DDT) and its breakdown product *p,p´*-dichlorodiphenyldichloroethylene (DDE). In contrast to PBDEs, DDT has not been used in the United States since 1972 but was used in Mexico until 2000. Given the differences in historical use of these chemicals, we hypothesized that Mexican-American children living in California would have higher levels of PBDEs but lower levels of DDT/DDE than their Mexican counterparts. By comparing these two populations, we sought to better understand the influence immigration has on PBDE levels in California Latino children and the relative contributions of PBDE exposure *in utero* and during early childhood.

## Methods

*Participants and procedures.* Children in this binational investigation participated in two affiliated studies, one located in California [Center for the Health Assessment of Mothers and Children of Salinas (CHAMACOS)] and the other in Mexico (Proyecto Mariposa). Written consent for maternal and child participation was obtained from all mothers. Assent was obtained from the CHAMACOS 7-year-olds. Human subjects protocols for both studies were approved by institutional review boards at University of California, Berkeley, and for Proyecto Mariposa, at the National Institute of Public Health in Mexico.

Detailed methods about the CHAMACOS study are described elsewhere (Eskenazi et al. 2003, 2004). Briefly, between 1999 and 2000, pregnant women were enrolled from prenatal clinics serving a low-income, Spanish-speaking farm worker population in the Salinas Valley in Monterey County, California. Women were eligible if they were ≤ 20 weeks gestation at enrollment, were ≥ 18 years of age, spoke Spanish or English, qualified to receive poverty-based government health insurance, and planned to deliver at the local county hospital. Of the 601 participants enrolled, 526 were followed to delivery of a live-born singleton infant. A total of 346 women provided a prenatal blood sample of sufficient volume for analysis. Structured interviews collecting demographic and other information were conducted in English or Spanish by bilingual, bicultural study staff during pregnancy and at various ages during childhood. Blood was collected when the children were 7 years of age (*n =* 339) and occurred between March 2007 and November 2008. For the present analysis, we excluded twins (*n =* 6), children lacking 7-year PBDE measurements (*n =* 59), and children whose mothers were not born in Mexico or of Mexican ancestry (*n =* 8). In addition, two children were missing 7-year lipid measurements. The final sample size was 264. Seven-year-old children with missing chemical measurements were not appreciably different in sociodemographic characteristics, including the length of time their mothers had lived in the United States, from those included in these analyses. There were 160 mother–child pairs with serums analyzed for PBDEs and organochlorine (OC) pesticides. We found no statistical difference in demographic characteristics between these 160 mother–child pairs and those without maternal blood (*n =* 104) except that the latter mothers had lived in the United States longer. Additionally, there were no differences in child PBDE or DDT/DDE levels between the two groups.

Detailed methods for Proyecto Mariposa have been described previously (Rosas et al. 2009). We recruited a low-income population in Mexico that was demographically similar to the CHAMACOS population. In both populations, the children had access to health care and were receiving government benefits for nutrition. In Mexico, between May and June 2006, we recruited a convenience sample of women and their 5-year-old children from government-run community health clinics serving families enrolled in the social welfare program Oportunidades. We enrolled participants from high-migration communities in the states of Michoacán, Guanajuato, and Jalisco, the states from which most of the CHAMACOS women originated.

Women and their children were eligible to participate if their child was approximately 5 years of age, the mother spoke fluent Spanish and was at least 18 years of age when her child was born, and the mother and child were currently receiving Oportunidades benefits, had lived exclusively in Mexico, and had not been to the United States for longer than 1 month. Of 317 families in the Proyecto Mariposa sample, 283 provided birth and demographic information and child blood samples of sufficient volume to perform chemical measurements. In Proyecto Mariposa, women were interviewed in Spanish by trained interviewers using a structured questionnaire similar to that used for CHAMACOS, although information about early childhood and pregnancy was obtained retrospectively for Proyecto Mariposa.

*Blood collection and chemical analysis.* Maternal blood samples were collected from the CHAMACOS women around the 26th week of gestation. Child blood samples were collected from the CHAMACOS and Proyecto Mariposa cohorts at the time of the interview and were immediately processed, with the sera stored at –80°C until shipment on dry ice to the U.S. Centers for Disease Control and Prevention (CDC; Atlanta, GA), where they were analyzed. PBDEs and OC pesticides were measured in serum using gas chromatography/isotope dilution high-resolution mass spectrometry (Sjödin et al. 2004). Samples were analyzed for 10 tri- to hepta-brominated congeners, BDEs 17, 28, 47, 66, 85, 99, 100, 153, 154, and 183 and for OC pesticides including DDT and DDE. Total lipids were determined based on the measurement of triglycerides and total cholesterol in serum using standard enzymatic methods (Roche Chemicals, Indianapolis, IN, USA) (Phillips et al. 1989).

*Data analysis.* Statistical analyses were conducted using Stata for Windows, version 10.1 (StataCorp LP, College Station, TX, USA).

We compared demographic characteristics for the two populations using *t*-tests for continuous and chi-square tests for categorical characteristics. Breast-feeding duration was censored at age 24 months. We calculated *z*-scores for children’s body mass index (BMI) (kilograms per square meter) using sex-specific BMI-for-age percentile data issued in 2000 by the CDC (National Center for Health Statistics 2005). CHAMACOS mothers’ length of time living in the United States at pregnancy was examined both as a continuous and as a categorical variable (≤ 1 year, 2–5 years, 6–10 years, or 11 years to lifetime).

Concentrations of PBDEs and OC pesticides were expressed on a blood lipid basis. PBDE congeners with < 65% detection frequency in both populations (BDEs 17, 66, and 183) were not included in these analyses. Values below the limit of detection (LOD) for which a signal was detected were coded with the concentration obtained; when no signal was detected, values were coded as the lowest concentration obtained for that congener divided by the square root of two (Hornung and Reed 1990). To compare PBDE levels between groups, we examined individual congeners and also calculated a direct sum of the seven congeners with at least 65% detected in either of the two populations (BDEs 28, 47, 85, 99, 100, 153, 154).

We transformed the individual and summed PBDE and OC pesticide concentrations to the log_10_ scale to approximate a normal distribution. We used *t*-tests to compare GMs and Pearson correlations to examine the correlations among PBDE congeners. We also employed Pearson correlations and GMs to examine relationships between serum concentrations for the CHAMACOS mother–child pairs.

We calculated for both the CHAMACOS and Proyecto Mariposa cohorts the unadjusted beta coefficients for the association between exposure concentrations (PBDEs, DDT, and DDE) and child’s breast-feeding duration.

We determined the unadjusted and adjusted beta coefficients and GMs and 95% confidence intervals (CIs) for children’s PBDEs (BDEs 47, 99, 100, and 153), DDT, and DDE concentrations by the mothers’ duration (years) living in the United States at the time of pregnancy. The mothers’ time living in the United States was examined as both a continuous variable (left-censored at 18 years) and as a categorical variable. Proyecto Mariposa mothers lived in Mexico for their lifetime, that is, 0 years in the United States. The multivariable models were adjusted for the child’s BMI *z*-score, breast-feeding duration, and maternal parity (parous or not). *p*-Values ≤ 0.05 were considered statistically significant.

## Results

Slightly more children in both cohorts were girls. CHAMACOS mothers were more educated than Proyecto Mariposa mothers, with about 20% versus < 1% having at least a high school education. CHAMACOS mothers were more likely to be multiparous and were slightly younger at delivery. Although almost all children in both cohorts had been breast-fed, CHAMACOS mothers breast-fed the index child a shorter period of time (mean = 8.9 months) than Proyecto Mariposa mothers (mean = 11.3 months). CHAMACOS children were heavier than Proyecto Mariposa children, as reflected in the BMI *z*-score adjusted for age and sex [see Supplemental Material, Table 1 (http://dx.doi.org/10.1289/ehp.1002874)].

**Table 1:** Distributions of concentrations of individual PBDE congeners and their sum by lipid-weight values (nanograms per gram lipid) in serum for Mexican-American children from the CHAMACOS study (*n *=**264; age 7 years) and Mexican children from Proyecto Mariposa (*n *=**283; age 5 years).

Study population	Percent > LOD	LOD range	Serum concentrations (ng/g lipid)												
			Congener	Minimum	10th	25th	50th	75th	90th	Maximum	GM (95% CI)	
BDE-28		CHAMACOS		89		0.3–5.6		< LOD		0.6		1.3		2.1		3.5		5.6		22.5		1.8	(1.6–2.1)
		Mariposa		20		0.3–4.5		< LOD		< LOD		< LOD		0.2		0.4		0.9		11.1		0.2	(0.2– 0.2)
BDE-47		CHAMACOS		100		0.4–8.0		1.9		17.4		27.7		46.9		76.8		136.0		582.0		47.1	(42.6–52.0)
		Mariposa		92		0.8–12.1		0.3		1.8		2.8		4.8		8.8		21.7		260.0		5.6	(4.9–6.4)
BDE-85		CHAMACOS		69		0.3–5.6		< LOD		0.3		0.6		0.9		1.6		2.6		13.5		0.9	(0.8–1.0)
		Mariposa		13		0.3–4.5		< LOD		< LOD		0.1		0.1		0.3		0.6		6.6		0.2	(0.2– 0.2)
BDE-99		CHAMACOS		100		0.3–5.6		0.4		3.7		6.2		10.6		19.3		36.8		193.0		11.1	(9.9–12.3)
		Mariposa		73		0.5–7.3		< LOD		0.5		0.8		1.4		2.9		7.0		135.0		1.6	(1.4–1.9)
BDE-100		CHAMACOS		100		0.3–5.6		0.8		3.9		5.9		10.8		16.5		32.4		127.0		10.6	(9.7– 11.7)
		Mariposa		92		0.3–4.5		< LOD		0.5		0.9		1.5		2.5		6.4		63.0		1.6	(1.4–1.8)
BDE-153		CHAMACOS		100		0.3–5.6		0.9		4.9		6.8		11.5		19.9		31.6		95.7		12.0	(10.9–13.1)
		Mariposa		99		0.3–4.5		0.4		1.0		1.4		2.1		3.7		7.0		52.3		2.4	(2.2–2.6)
BDE-154		CHAMACOS		79		0.3–5.6		< LOD		0.4		0.7		1.1		1.9		3.5		14.1		1.2	(1.1–1.3)
		Mariposa		17		0.3–4.5		< LOD		< LOD		0.1		0.2		0.4		0.7		13.4		0.2	(0.2– 0.2)
ΣPBDE*a*		CHAMACOS		—		—		6.3		33.3		52.9		89.1		135.0		236.9		1,011.1		87.8	(80.0–96.3)
		Mariposa		—		—		1.1		4.4		6.4		10.7		19.0		45.7		487.3		12.3	(11.0–13.8)
10th, 25th, 50th, 75th, and 90th are percentiles.**a**Comprising BDEs 28, 47, 85, 99, 100, 153, and 154.																							

Of the 10 congeners, seven had > 65% detection (BDEs 28, 47, 85, 99, 100, 153, and 154) in the CHAMACOS population, compared with only four congeners (BDEs 47, 99, 100, and 153) in the Proyecto Mariposa population. Table 1 presents the levels and distributions for the serum concentrations of these seven PBDE congeners and their sums for the CHAMACOS and Proyecto Mariposa children. GMs, medians, and the 90th percentiles for all seven PBDE congeners were higher in CHAMACOS than Proyecto Mariposa children. The GM (95% CI) of the sum of the seven congeners (ΣPBDE) was 87.8 (80.0–96.3) ng/g lipid for CHAMACOS versus 12.3 (11.0–13.8) ng/g lipid for Proyecto Mariposa children. The concentration of ΣPBDE exceeded 100 ng/g lipid for 42% of CHAMACOS children compared with only 4% of Proyecto Mariposa children. The seven congener concentrations were highly correlated within both groups (CHAMACOS: *r* = 0.46–0.97; *p* < 0.0001; Proyecto Mariposa: *r* = 0.57–0.96; *p* < 0.0001).

PBDEs were measured in blood collected at age 5 years as well as at age 7 years for eight CHAMACOS children. We found that the levels at 5 years of age were slightly higher; specifically, for the ΣPBDE, the GM (95% CI) was 188.4 (78.6–451.9) ng/g lipid at 5 years and 128.2 (66.5–247.0) ng/g lipid at 7 years (paired *t*-test *p* = 0.10).

Mean blood concentrations of ΣPBDE in CHAMACOS mothers during their pregnancy were significantly lower than levels measured 7 years later in their children [GM (95% CI) = 25.0 (21.5–29.0) ng/g lipid vs. 85.8 (76.2–96.6) in their children (paired *t*-test *p* = < 0.001)] and were moderately correlated (*r* = 0.25; *p* = 0.002). The median, 75th percentile and 90th percentile blood concentrations of ΣPBDE in CHAMACOS children at age 7 years were three or more times higher than those of their mothers at 26 weeks of gestation (data not shown).

For the CHAMACOS cohort, length of time the child breast-fed was positively associated with the child’s serum concentrations of BDE-47 [percent change in concentration per month breast fed (95% CI) = 1.1% (–0.2 to 2.4); *p* = 0.10)], BDE-99 [1.4% (–0.1 to 2.9); *p =* 0.06], BDE-100 [0.9% (–0.4 to 2.2); *p =* 0.19], BDE-153 [0.7% (–0.5 to 1.9); *p =* 0.23], and ΣPBDE [1.0% (–0.2 to 2.3); *p =* 0.12], albeit not significantly. With the exception of BDE-153 [2.0% (0.6–3.4); *p =* 0.004], associations with breast-feeding duration in the Proyecto Mariposa cohort were even weaker than for the CHAMACOS cohort, that is, BDE-47: 0.5% (–1.3 to 2.3), *p =* 0.62; BDE-99: 0.8% (–1.2 to 2.8); *p =* 0.42; BDE-100: 0.7% (–1.0 to 2.4); *p =* 0.41; and ΣPBDE: 0.9% (–0.8 to 2.6); *p =* 0.29.

**Figure 1:**
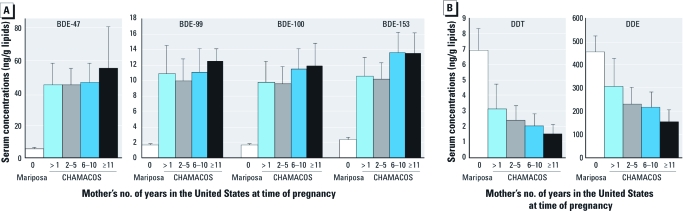
GMs (adjusted for parity, breast-feeding duration, and child BMI *z*-score) and 95% CIs for PBDE congeners (*A*) and DDT and DDE (*B*) in child serum by length of time mother lived in the United States at time of pregnancy (CHAMACOS children at 7 years, Proyecto Mariposa children at 5 years).

Within the CHAMACOS cohort, the child’s ΣPBDE serum concentration increased 1.8% (95% CI, 0.2–3.5%; *p =* 0.03) for each year his or her mother resided in the United States, after adjusting for breast-feeding duration, parity and child’s BMI *z*-score. We also observed similar positive relationships for length of U.S. residence and the concentrations of individual congeners [BDE-47: 1.8% (0.1–3.6), *p =* 0.04; BDE-99: 1.6% (–0.2 to 3.6), *p =* 0.09; BDE-100: 1.8% (0.2–3.4), *p =* 0.05; and BDE-153: 2.1% (0.7–3.5), *p =* 0.004]. There was no appreciable difference when the results were not adjusted for breast-feeding and other covariates. In Figure 1A, adjusted GMs for PBDE levels in children are shown for the Proyecto Mariposa cohort and according to categories of mother’s length of time living in the United States at pregnancy for the CHAMACOS cohort. For all congeners, children born in the United States had significantly higher levels than children born in Mexico, regardless of the length of the maternal residence in the United States (*p* < 0.001), even for the children whose mothers had lived in the United States ≤ 1 year at the time of pregnancy.

Table 2 presents serum concentrations for DDT and DDE. In contrast to PBDEs, the serum concentrations for DDT and DDE for CHAMACOS children were considerably lower than those for Proyecto Mariposa children. Only 20% of CHAMACOS children had DDT serum levels > LOD, compared with 69% of Proyecto Mariposa children. Even though DDE levels were > LOD for all children in both studies, the unadjusted GMs of DDE concentrations were more than twice as high for the Mexican than for the Mexican-American children [GM = 461.9 (396.6–538.0) ng/g lipid vs. 215.2 (185.9–249.0) ng/g lipid] and the 95% CIs for the two groups did not overlap (*p* < 0.001). In the CHAMACOS cohort, maternal and child DDT and DDE levels were strongly correlated (DDE *r =* 0.63, DDT *r =* 0.71, *p* < 0.001, *n =* 230). In contrast to the pattern observed for PBDEs, the median, 75th, and 90th percentiles of maternal serum concentrations during pregnancy were six or more times higher for DDE and DDT than the children’s serum concentrations at 7 years (data not shown). Children’s DDE and DDT levels were related to the length of time breast-fed for the CHAMACOS children [percent change in concentration per month breast-fed (95% CI) = DDE: 9.6% (7.8–11.4), *p =* < 0.001; DDT: 5.3% (3.5–7.1), *p =* < 0.001] but less so for the Proyecto Mariposa cohort [DDE: 3.4% (1.2–5.7), *p =* 0.002; DDT: 1.8% (–1.2 to 4.9), *p =* 0.24].

**Table 2:** Distributions of serum concentrations of *p,p*´-DDT and *p,p*´-DDE in Mexican-American children from the CHAMACOS study (*n *=**264; age 7 years) and Mexican children from Proyecto Mariposa (*n *=**283; age 5 years).

Study population	Percent > LOD	LOD range	Serum concentrations (ng/g lipid)												
			Chemical	Minimum	10th	25th	50th	75th	90th	Maximum	GM (95% CI)	
DDT		CHAMACOS		20		1.4–27.8		< LOD		0.8		1.1		1.6		2.8		6.9		1,430		2.1	(1.8–2.4)
		Mariposa		69		1.4–22.7		< LOD		1.6		2.5		3.8		8.7		86.7		3,700		7.0	(5.6–8.6)
DDE		CHAMACOS		100		2.4–49.0		25.2		62.5		95.2		163.0		392.5		1,190		23,300		215.2	(185.9–249.0)
		Mariposa		100		2.5–40.0		49.2		121.0		200.0		357.0		741.0		2,440		34,500		461.9	(396.6–538.0)
10th, 25th, 50th, 75th, and 90th are percentiles.																							

Among CHAMACOS children, the length of time the mother had lived in the United States (continuous variable) was inversely related to the child’s serum concentrations of DDE and DDT adjusted for breast-feeding duration, parity, and BMI *z*-score [percent change in concentration per year in the United States (95% CI) = DDE: –2.5% (–4.5 to –0.5), *p =* 0.02; DDT: –3.6 (–5.7 to –1.4), *p =* 0.001] (Figure 1B). The children whose mothers had lived in the United States for even ≤ 1 year at the time of pregnancy had lower levels of DDT (*p* = 0.002) and somewhat lower levels of DDE (*p =* 0.12) than the Mexican children.

## Discussion

We found that PBDE serum concentrations in a cohort of California Mexican-American school-age children were three times higher than concentrations in their mothers during pregnancy (about 7 years earlier) and seven times higher than those in children living in Mexico. The differences between PBDE levels in the two childhood cohorts were found regardless of whether we controlled for duration of breast-feeding, an important source of PBDE exposure in young children (Daniels et al. 2010; Hooper et al. 2007). The fact that the CHAMACOS children whose mothers lived in the United States at the time of pregnancy for even ≤ 1 year had higher levels than Mexican children supports the hypothesis that most of the body burden measured in the CHAMACOS 7-year-olds was not a residual of breast-feeding. Instead, the principal sources of children’s exposure were likely house dust and food (Frederiksen et al. 2009; Lorber 2008).

In contrast to the pattern we observed for PBDEs, DDT/DDE levels in the children decreased with the time their mothers had lived in the United States; even children whose mothers had lived in the United States ≤ 1 year had lower levels than Mexican children. DDT has been banned in the United States since the 1970s but was used in parts of Mexico until 2000. Therefore, the exposure of CHAMACOS children to DDT likely occurred *in utero* or from breast-feeding, with little environmental (Hwang et al. 2008) or nonlactational dietary (Food and Drug Administration 2002) exposure to DDT while living in the United States, whereas in Mexico there may have been continued environmental exposure. This hypothesis is supported by the stronger relationship between breast-feeding and DDT/DDE levels in the CHAMACOS cohort than in Proyecto Mariposa.

We also observed PBDE concentrations in CHAMACOS children that were three times higher than in their mothers. The higher levels in children than adults are consistent with results from other studies. For example, in a case report of a northern California family, Fischer et al. (2006) reported PBDE levels in the children two to five times higher than those of their parents. A recent investigation of 20 U.S. mothers and their 1.5- to 4-year-olds found that PBDE concentrations were about 2.8 times higher in the children than in their mothers (blood samples were collected at the same time) (Lunder et al. 2010). Higher levels of PBDE in children may be attributable to greater frequency of hand-to-mouth contact among children compared with adults (Stapleton and Dodder 2008), a pattern consistent with exposure routes for lead, another persistent toxicant for which nondietary ingestion in dust is a primary exposure route (Dixon et al. 2009). However, unlike these previous studies, the maternal and child blood samples in CHAMACOS were not collected concurrently (maternal blood was collected during pregnancy and children’s blood was collected approximately 7 years later). Thus, we cannot rule out that the higher levels observed in the children are due at least partly to differences in the years of blood collection. Although penta-PBDEs were phased out after 2004, exposure likely continued, given the ongoing release of PBDEs from aging materials produced before the phase-out. These PBDEs potentially accumulated and persisted in environmental media, resulting in higher contamination and exposure by the time of the later blood collection of the children.

**Figure 2:**
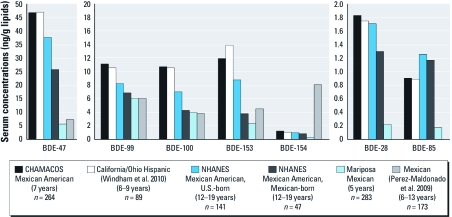
Unadjusted GMs of serum concentrations of PBDE congeners in Mexican-American children in CHAMACOS and Mexican children in Proyecto Mariposa compared with concentrations from other cohorts of Latino children.

The few studies of PBDEs in children’s blood worldwide indicate that children living in California have some of the highest documented PBDE serum concentrations. Figure 2 presents concentrations measured in Mexican and Mexican-American children. Except for BDE-153, the blood concentrations for the penta-BDE congeners in the CHAMACOS cohort are similar to those reported by Windham et al. (2010) (Windham G, personal communication) for 89 Hispanic girls, the vast majority of whom were California residents (*n =* 80). PBDE levels in the CHAMACOS children were higher than in a representative sample of Mexican-American children (12–19 years old) born in the United States and even higher than those born in Mexico (NHANES) for all congeners except BDE-85 (Sjödin et al. 2008b). The PBDE levels in CHAMACOS children were not only higher than in Mexican children in Proyecto Mariposa, but also higher (for all congeners except BDE-154) than in children 6–13 years of age from six other areas of Mexico, including those children living in communities selected because of proximity to landfills or industry (e.g., an electronics assembly plant or brick kilns) (Perez-Maldonado et al. 2009). To date, the PBDE levels measured in California children are exceeded only by levels reported for a study of children living and working on hazardous waste sites in Nicaragua (Athanasiadou et al. 2008).

As noted by Zota et al. (2008), high levels of flame retardants in California homes and residents may be an unintended consequence of government regulation. California has unique furniture flammability standards, the most important of which is Technical Bulletin 117 (TB 117), which was promulgated in the mid-1970s (California Bureau of Electronic and Appliance Repair 2000). The enforcement of TB 117 has resulted in the addition of millions of pounds of flame retardants to polyurethane foam used in seating furniture and infant products, such as car and other transportation seats, strollers, and carpet pads. Until 2005, the predominant chemical flame retardant used to comply with TB 117 was penta-BDE. The PBDE congeners analyzed in this study are all found in penta-BDE, with BDE-47 and BDE-99 the most common congeners by weight. Because these compounds are semivolatile and are not chemically bound to substrates, they can migrate into the indoor environment, particularly into house dust. This may explain why the levels of penta-BDE congeners are 7 to 10 times higher in house dust collected in California versus other parts of the United States and Canada (Zota et al. 2008). House dust exposures may explain elevated PBDE levels in children, given their proximity to the floor and frequent hand-to-mouth behaviors (Stapleton et al. 2008).

This investigation has several strengths. It is one of the largest studies of PBDE exposures in children in the world and the largest of Latino children. We have conducted the first binational study of PBDEs in comparable populations in the United States (CHAMACOS) and Mexico (Proyecto Mariposa) and with similar methods of data collection and analyses. The Proyecto Mariposa provides some of the first data on PBDE levels in Mexican children, as only one previous smaller study has been conducted on children who were primarily selected for high exposure (Perez-Maldonado et al. 2009). In addition, CHAMACOS is the only longitudinal study of PBDEs in children followed from the *in utero* period to childhood, and thus allowed for the examination of both prenatal and childhood exposure.

This report also has several limitations. Ideally, we would have liked to compare PBDE levels obtained from the two populations at the same ages. Nevertheless, we were able to measure PBDEs in samples from eight children at both 5 and 7 years of age, and found that the concentrations were only slightly higher when the children were younger. This observation is in accordance with that of Toms et al. (2009), who found that Australian children 2.6–3 years of age had the highest blood concentrations relative to those of infants, older children, and adults, which suggested the possibility that PBDE body burdens may decline after early childhood. Thus, we may have slightly underestimated the differences between the Mexican-American and Mexican cohorts because of the younger age of the Mexican cohort. Another limitation of this study is that we did not measure some of the higher-brominated compounds such as BDE-209. However, in a study of California children, BDE-209 represented a very small fraction of the total blood concentrations of PBDEs (Rose et al. 2010).

CHAMACOS comprises a unique population of Mexican-American children who have elevated serum concentrations of PBDEs by virtue of living in California and were born and breast-fed from mothers with high levels of DDT/DDE because they emigrated from Mexico (Bradman et al. 2007). In addition, as is true of more than a quarter of Latino children living in California, the CHAMACOS participants live in poverty (Annie E. Casey Foundation 2003). Zota et al. (2008, 2010) have suggested that PBDE levels may be higher in lower-income homes because of the presence of poorly manufactured furniture, deteriorated PBDE-treated furniture foam, and poorer ventilation. We recently reported (Quirós-Alcalá et al. 2011) that the median levels of PBDEs in dust collected from 20 homes of Mexican immigrants in Salinas and in Oakland were up to 20 times higher than those found in homes elsewhere in the United States (Quirós-Alcalá et al. 2011; Stapleton et al. 2005; Zota et al. 2008). The maximum PBDE concentrations in the Oakland urban homes were the highest reported to date in the United States and much higher than those reported from Europe and Asia (Harrad et al. 2008; Tan et al. 2007).

Given the growing evidence documenting potential health effects of PBDE exposure (Chevrier et al. 2010; Harley et al. 2010; Herbstman et al. 2010; Roze et al. 2009), the levels in young children noted in this study present a major public health challenge. Although this challenge is particularly pronounced for California children, it is also relevant to other regions in the United States, where exposures are increased through manufacturing practices seeking to achieve TB 117 compliance even in products not destined for California. This practice has resulted in the dissemination of penta-BDE-containing products throughout the United States. Some penta-BDE substitutes have recently been detected with high frequency in furniture foam and house dust collected outside of California (Stapleton et al. 2009), suggesting the potential for ongoing flame retardant exposures in homes and offices throughout the United States, due at least in part to rules developed in California over three decades ago. With the legislative ban of penta-BDE in products sold in California, other halogenated flame retardants, including chlorinated organophosphates and a variety of proprietary mixtures containing halogenated aromatic compounds, have been used instead to comply with TB 117. Thus, children’s exposure to organohalogen flame retardant chemicals used in furniture will continue given current regulations and will need to be documented. In addition, the toxicology and health consequences of chemical replacements to penta-BDE should be investigated and weighed against their purported fire safety benefits.

## Supplemental Material

(72 KB) PDF

## References

[r1] Akutsu K, Takatori S, Nozawa S, Yoshiike M, Nakazawa H, Hayakawa K (2008). Polybrominated diphenyl ethers in human serum and sperm quality.. Bull Environ Contam Toxicol.

[r2] Annie E. Casey Foundation (2003). Kids Count: Latino Children Pocket Guide: State-Level Measures of Child Well-Being From the 2000 Census.

[r3] Athanasiadou M, Cuadra SN, Marsh G, Bergman A, Jakobsson K (2008). Polybrominated diphenyl ethers (PBDEs) and bioaccumulative hydroxylated PBDE metabolites in young humans from Managua, Nicaragua.. Environ Health Perspect.

[r4] Bradman A, Fenster L, Sjodin A, Jones RS, Patterson DG, Eskenazi B (2007). Polybrominated diphenyl ether levels in the blood of pregnant women living in an agricultural community in California.. Environ Health Perspect.

[r5] California Bureau of Electronic and Appliance Repair, Home Furnishings and Thermal Insulation (BEARHFTI) (2000). Technical Bulletin 117: Requirements, Test Procedure and Apparatus for Testing the Flame Retardance of Resilient Filling Materials Used in Upholstered Furniture.. http://www.bhfti.ca.gov/industry/117.pdf.

[r6] California Perinatal Profiles (2009). Using Perinatal Profiles Data for Quality Improvement: Perinatal Profiles of California 2007 and 5-Year Cohort Data.. https://perinatalprofiles.berkeley.edu/PublicDocuments/QIGuide/QIguide.pdf.

[r7] Chao HR, Shy CG, Wang SL, Chih-Cheng Chen S, Koh TW, Chen FA (2010). Impact of non-occupational exposure to polybrominated diphenyl ethers on menstruation characteristics of reproductive-age females.. Environ Int.

[r8] Chevrier J, Harley K, Bradman A, Gharbi M, Sjödin A, Eskenazi B. (2010). Polybrominated diphenylether (PBDE) flame retardants and thyroid hormone during pregnancy.. Environ Health Perspect.

[r9] Daniels JL, Pan IJ, Jones R, Anderson S, Patterson DG, Needham LL (2010). Individual characteristics associated with PBDE levels in U.S. human milk samples.. Environ Health Perspect.

[r10] Dixon SL, Gaitens JM, Jacobs DE, Strauss W, Nagaraja J, Pivetz T (2009). Exposure of U.S. children to residential dust lead, 1999–2004. II. The contribution of lead-contaminated dust to children’s blood lead levels.. Environ Health Perspect.

[r11] Eskenazi B, Bradman A, Gladstone EA, Jaramillo S, Birch K, Holland NT (2003). CHAMACOS, a longitudinal birth cohort study: lessons from the fields.. J Children’s Health.

[r12] Eskenazi B, Harley K, Bradman A, Weltzien E, Jewell NP, Barr DB (2004). Association of *in utero* organophosphate pesticide exposure and fetal growth and length of gestation in an agricultural population.. Environ Health Perspect.

[r13] Fischer D, Hooper K, Athanasiadou M, Athanassiadis I, Bergman A. (2006). Children show highest levels of polybrominated diphenyl ethers in a California family of four: a case study.. Environ Health Perspect.

[r14] Food and Drug Administration (2002). Food and Drug Administration Pesticide Program: Residue Monitoring 2002. Silver Spring, MD:Food and Drug Administration.. http://www.fda.gov/downloads/Food/FoodSafety/FoodContaminantsAdulteration/Pesticides/ResidueMonitoringReports/ucm126088.pdf.

[r15] Frederiksen M, Vorkamp K, Thomsen M, Knudsen LE (2009). Human internal and external exposure to PBDEs—a review of levels and sources.. Int J Hyg Environ Health.

[r16] Geyer HJ, Schramm KW, Darnerud PO, Aune M, Feicht A, Fried KW (2004). Terminal elimination half-lives of the brominated flame retardants TBBPA, HBCD, and lower brominated PBDEs in humans.. Organohalogen Compounds.

[r17] Harley KG, Marks AR, Chevrier J, Bradman A, Sjodin A, Eskenazi B (2010). PBDE concentrations in women’s serum and fecundability.. Environ Health Perspect.

[r18] Harrad S, Ibarra C, Diamond M, Melymuk L, Robson M, Douwes J (2008). Polybrominated diphenyl ethers in domestic indoor dust from Canada, New Zealand, United Kingdom and United States.. Environ Int.

[r19] Herbstman JB, Sjodin A, Apelberg BJ, Witter FR, Halden RU, Patterson DG (2008). Birth delivery mode modifies the associations between prenatal polychlorinated biphenyl (PCB) and polybrominated diphenyl ether (PBDE) and neonatal thyroid hormone levels.. Environ Health Perspect.

[r20] Herbstman JB, Sjodin A, Kurzon M, Lederman SA, Jones RS, Rauh V (2010). Prenatal exposure to PBDEs and neurodevelopment.. Environ Health Perspect.

[r21] Hites RA (2004). Polybrominated diphenyl ethers in the environment and in people: a meta-analysis of concentrations.. Environ Sci Technol.

[r22] Hooper K, She J, Sharp M, Chow J, Jewell N, Gephart R (2007). Depuration of polybrominated diphenyl ethers (PBDEs) and polychlorinated biphenyls (PCBs) in breast milk from California first-time mothers (primiparae).. Environ Health Perspect.

[r23] Hornung RW, Reed LD (1990). Estimation of average concentration in the presence of nondetectable values.. Appl Occup Environ Hyg.

[r24] Hwang HM, Park EK, Young TM, Hammock BD (2008). Occurrence of endocrine-disrupting chemicals in indoor dust.. Sci Total Environ.

[r25] Johnson PI, Stapleton HM, Sjodin A, Meeker JD (2010). Relationships between polybrominated diphenyl ether concentrations in house dust and serum.. Environ Sci Technol.

[r26] Johnson-Restrepo B, Kannan K. (2009). An assessment of sources and pathways of human exposure to polybrominated diphenyl ethers in the United States.. Chemosphere.

[r27] Lorber M. (2008). Exposure of Americans to polybrominated diphenyl ethers.. J Expo Sci Environ Epidemiol.

[r28] Lunder S, Hovander L, Athanassiadis I, Bergman A. (2010). Significantly higher polybrominated diphenyl ether levels in young U.S. children than in their mothers.. Environ Sci Technol.

[r29] Meeker JD, Johnson PI, Camann D, Hauser R (2009). Polybrominated diphenyl ether (PBDE) concentrations in house dust are related to hormone levels in men.. Sci Total Environ.

[r30] National Center for Health Statistics (2005). CDC Growth Charts, United States.. http://www.cdc.gov/growthcharts.

[r31] Perez-Maldonado IN, Ramirez-Jimenez Mdel R, Martinez-Arevalo LP, Lopez-Guzman OD, Athanasiadou M, Bergman A (2009). Exposure assessment of polybrominated diphenyl ethers (PBDEs) in Mexican children.. Chemosphere.

[r32] Phillips DL, Pirkle JL, Burse VW, Bernert JT, Henderson LO, Needham LL (1989). Chlorinated hydrocarbon levels in human serum: effects of fasting and feeding.. Arch Environ Contam Toxicol.

[r33] Quirós-Alcalá L, Bradman A, Nishioka M, Harnly ME, Hubbard A, McKone TE (2011). Concentrations and loadings of polybrominated diphenyl ethers in dust from low-income households in California.. Environ Int.

[r34] Rosas LG, Harley K, Fernald LC, Guendelman S, Mejia F, Neufeld LM (2009). Dietary associations of household food insecurity among children of Mexican descent: results of a binational study.. J Am Diet Assoc.

[r35] Rose M, Bennett DH, Bergman A, Fangstrom B, Pessah IN, Hertz-Picciotto I (2010). PBDEs in 2-5 year-old children from California and associations with diet and indoor environment.. Environ Sci Technol.

[r36] Roze E, Meijer L, Bakker A, Van Braeckel KN, Sauer PJ, Bos AF (2009). Prenatal exposure to organohalogens, including brominated flame retardants, influences motor, cognitive, and behavioral performance at school age.. Environ Health Perspect.

[r37] Sjödin A, Jones RS, Lapeza CR, Focant JF, McGahee EE, Patterson DG (2004). Semiautomated high-throughput extraction and cleanup method for the measurement of polybrominated diphenyl ethers, polybrominated biphenyls, and polychlorinated biphenyls in human serum.. Anal Chem.

[r38] Sjödin A, Papke O, McGahee E, Focant JF, Jones RS, Pless-Mulloli T (2008a). Concentration of polybrominated diphenyl ethers (PBDEs) in household dust from various countries.. Chemosphere.

[r39] Sjödin A, Wong LY, Jones RS, Park A, Zhang Y, Hodge C (2008b). Serum concentrations of polybrominated diphenyl ethers (PBDEs) and polybrominated biphenyl (PBB) in the United States population: 2003–2004.. Environ Sci Technol.

[r40] Stapleton HM, Dodder NG (2008). Photodegradation of decabromodiphenyl ether in house dust by natural sunlight.. Environ Toxicol Chem.

[r41] Stapleton HM, Dodder NG, Offenberg JH, Schantz MM, Wise SA (2005). Polybrominated diphenyl ethers in house dust and clothes dryer lint.. Environ Sci Technol.

[r42] Stapleton HM, Kelly SM, Allen JG, McClean MD, Webster TF (2008). Measurement of polybrominated diphenyl ethers on hand wipes: estimating exposure from hand-to-mouth contact.. Environ Sci Technol.

[r43] Stapleton HM, Klosterhaus S, Eagle S, Fuh J, Meeker JD, Blum A (2009). Detection of organophosphate flame retardants in furniture foam and U.S. house dust.. Environ Sci Technol.

[r44] Tan J, Cheng SM, Loganath A, Chong YS, Obbard JP (2007). Polybrominated diphenyl ethers in house dust in Singapore.. Chemosphere.

[r45] Toms LM, Sjödin A, Harden F, Hobson P, Jones R, Edenfield E (2009). Serum polybrominated diphenyl ether (PBDE) levels are higher in children (2–5 years of age) than in infants and adults.. Environ Health Perspect.

[r46] Turyk ME, Persky VW, Imm P, Knobeloch L, Chatterton R, Anderson HA (2008). Hormone disruption by PBDEs in adult male sport fish consumers.. Environ Health Perspect.

[r47] U.S. Census Bureau (2009). American Community Survey (ACS): Public Use Microdata Sample (PUMS) 2008 1-Year.. http://factfinder.census.gov/home/en/acs_pums_2008_1yr.html.

[r48] Windham GC, Pinney SM, Sjodin A, Lum R, Jones RS, Needham LL (2010). Body burdens of brominated flame retardants and other persistent organo-halogenated compounds and their descriptors in US girls.. Environ Res.

[r49] Wu N, Herrmann T, Paepke O, Tickner J, Hale R, Harvey LE (2007). Human exposure to PBDEs: associations of PBDE body burdens with food consumption and house dust concentrations.. Environ Sci Technol.

[r50] Zota AR, Adamkiewicz G, Morello-Frosch RA (2010). Are PBDEs an environmental equity concern? Exposure disparities by socioeconomic status.. Environ Sci Technol.

[r51] Zota AR, Rudel RA, Morello-Frosch RA, Brody JG (2008). Elevated house dust and serum concentrations of PBDEs in California: unintended consequences of furniture flammability standards?. Environ Sci Technol.

[r52] Zuurbier M, Leijs M, Schoeters G, ten Tusscher G, Koppe JG (2006). Children’s exposure to polybrominated diphenyl ethers.. Acta Paediatr.

