# Supplementary material for: A Comparison of PBDE Serum Concentrations in Mexican and Mexican-American Children Living in California
Source: Environ Health Perspect. 2011 Apr 15;119(10):1442–8. doi: 10.1289/ehp.1002874 (PMC3230428; doi:10.1289/ehp.1002874)
Supplement: (72 KB) PDF [file ehp.1002874.s001.pdf]

Supplemental Material: A comparison of PBDE serum concentrations in Mexican and Mexican-American children living in California

Brenda Eskenazi[1], Laura Fenster[1], Rosemary Castorina[1], Amy R. Marks[1], Andreas Sjödin[2], Lisa Goldman Rosas[1], Nina Holland[1], Armando Garcia Guerra[3], Lizbeth Lopez-Carillo[3], Asa Bradman[1]

[1] Center for Children's Environmental Health Research, School of Public Health, University of California, Berkeley, CA

[2] Division of Laboratory Sciences, National Center for Environmental Health, Centers for Disease Control and Prevention, Atlanta, GA,

[3] National Institute of Public Health, Cuernavaca, Mexico

Please address correspondence to:

Brenda Eskenazi, Ph.D.

Center for Children’s Environmental Health Research

School of Public Health

University of California at Berkeley

1995 University Avenue

Suite 265

Berkeley, California 94602

Supplemental Table 1. Demographic characteristics of CHAMACOS and Proyecto Mariposa mothers and children.

Characteristic	Mariposa n = 283	CHAMACOS n = 264	Statistic (p-value)
Mother			
Education, n(%)			
≤ 6th Grade	215 (76.0)	118 (44.7)	X^2= 79.6
7-12th Grade	66 (23.3)	93 (35.2)	(p<0.001)
≥ Graduate High School	2 (0.7)	53 (20.1)	
Years in U.S. at Pregnancy, n(%)			
≤1		51 (19.3)	
2-5		70 (26.5)	
6-10		75 (28.4)	
≥11		68 (25.8)	
Parity			
Yes	39 (13.8)	85 (32.2)	X^2= 26.4
No	244 (86.2)	179 (67.8)	(p <0.001)
Age at Delivery, M±SD	27.6±6.3	26.3±5.1	X^2= 7.2 (p=0.007)
Child			
Sex, n(%)			
Boy	131 (46.3)	122 (46.2)	X^2= 0.0
Girl	152 (53.7)	142 (53.8)	(p=1.0)
Age (years), M±SD	5.4±0.3	7.1±0.2	X^2= 409.0 (p<0.001)
Child Breastfed (months), M±SD	11.3±8.6	8.9±8.5	X^2= 16.6 (p<0.001)
BMI (kg/m²), M±SD*	15.6±1.8	18.9±3.9	X^2= 165.8 (p<0.001)
BMI (Z-score), M±SD**	0.0±1.7	1.1±1.0	X^2= 120.0 (p<0.001)

* Age at time of measurements were approximately 5 years for Proyecto Mariposa children and 7 years for CHAMACOS children.
**Adjusted for age and sex, www/cdc/growthcharts/zscore.htm
